# 3-{1-[2-(2-Chloro­phen­yl)hydrazinyl­idene]-2,2,2-trifluoro­eth­yl}-7-diethyl­amino-2*H*-chromen-2-one

**DOI:** 10.1107/S1600536811031916

**Published:** 2011-08-17

**Authors:** Hao Chen, Li Cai, Chaochao Yu, Hongqi Li

**Affiliations:** a Key Laboratory of Science & Technology of Eco-Textiles, Ministry of Education, College of Chemistry, Chemical Engineering & Biotechnology, Donghua University, Shanghai 201620, People’s Republic of China

## Abstract

The title compound, C_21_H_19_ClF_3_N_3_O_2_, has a structure related to other coumarin derivatives that have been used as fluorescent probes of metal ions. The dihedral angle between the coumarin ring system and the chlorobenzene ring is 42.99 (9)°. Intra­molecular hydrogen bonding occurs *via* N—H⋯O and N—H⋯Cl inter­actions, generating *S*(7) and *S*(5) rings, respectively. The crystal packing is stabilized by weak C—H⋯O hydrogen bonds.

## Related literature

For applications of coumarins and coumarin derivatives, see: Trenor *et al.* (2004 ▶); Starcevic *et al.* (2011 ▶); Danko *et al.* (2011 ▶). For the synthesis of the title compound and related structures, see: Li *et al.* (2011 ▶).
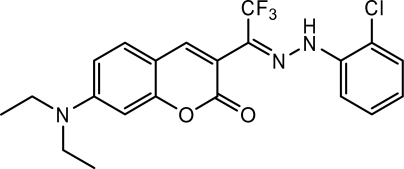


## Experimental

### 

#### Crystal data

C_21_H_19_ClF_3_N_3_O_2_
*M* *_r_* = 437.84
Orthorhombic, 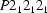

*a* = 7.940 (6) Å
*b* = 12.602 (9) Å
*c* = 20.233 (15) Å
*V* = 2025 (3) Å^3^
*Z* = 4
Mo *K*α radiation
μ = 0.24 mm^−1^
*T* = 296 K
0.20 × 0.20 × 0.18 mm

#### Data collection

Bruker APEXII CCD diffractometer
Absorption correction: multi-scan (*SADABS*; Bruker, 2001 ▶) *T*_min_ = 0.954, *T*_max_ = 0.958
10369 measured reflections
3576 independent reflections
3162 reflections with *I* > 2σ(*I*)
*R*_int_ = 0.023

#### Refinement

*R*[*F* ^2^ > 2σ(*F* ^2^)] = 0.032
*wR*(*F* ^2^) = 0.078
*S* = 1.03
3576 reflections
273 parameters
H-atom parameters constrained
Δρ_max_ = 0.15 e Å^−3^
Δρ_min_ = −0.21 e Å^−3^
Absolute structure: Flack (1983 ▶), 1511 Friedel pairs
Flack parameter: −0.09 (6)

### 

Data collection: *APEX2* (Bruker, 2003 ▶); cell refinement: *SAINT-Plus* (Bruker, 2003 ▶); data reduction: *SAINT-Plus*; program(s) used to solve structure: *SHELXS97* (Sheldrick, 2008 ▶); program(s) used to refine structure: *SHELXL97* (Sheldrick, 2008 ▶); molecular graphics: *SHELXTL* (Sheldrick, 2008 ▶); software used to prepare material for publication: *SHELXTL*.

## Supplementary Material

Crystal structure: contains datablock(s) global, I. DOI: 10.1107/S1600536811031916/lr2022sup1.cif

Structure factors: contains datablock(s) I. DOI: 10.1107/S1600536811031916/lr2022Isup2.hkl

Supplementary material file. DOI: 10.1107/S1600536811031916/lr2022Isup3.cml

Additional supplementary materials:  crystallographic information; 3D view; checkCIF report

## Figures and Tables

**Table 1:** Hydrogen-bond geometry (Å, °)

*D*—H⋯*A*	*D*—H	H⋯*A*	*D*⋯*A*	*D*—H⋯*A*
N3—H3*A*⋯Cl1	0.86	2.57	2.960 (2)	109
N3—H3*A*⋯O2	0.86	2.22	2.761 (3)	121
C14—H14⋯O2^i^	0.93	2.55	3.316 (3)	140

Symmetry code: (i) 
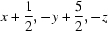
.

**Fig. 1.**
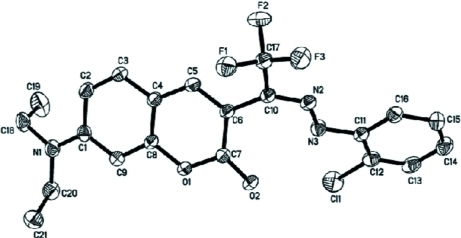
ORTEP plot of the title compound with displacement ellipsoids at the 30% probability level. H atoms are omitted for clarity.

## supplementary crystallographic information

### Comment

Because the structure of benzopyrone has many advantages including high fluorescence quantum yield, large Stokes shift, excellent light stability, and less toxicity coumairns have been widely used in the fields of biology, medicine (Starcevic *et al.*, 2011), perfumes, cosmetics (Trenor *et al.*, 2004), and fluorescent dyes (Danko *et al.*, 2011). We have synthesized a series of novel coumarin derivatives and found that one of them 3-(2-benzoylhydrazonotrifluoroethyl)-7- (*N*,*N*-diethylamino)coumarin can be used as fluorescent probes of Cu(II) and Ni(II) (Li *et al.*, 2011). Herein we report the single-crystal structure of 3-(2-Chlorophenylhydrazonotrifluoroethyl)-7-(N,*N*- diethylamino)coumarin, which may be a good candidate for fluorescent probe of metal ions.

### Experimental

The title compound was prepared as reported in the literature (Li *et al.*, 2011). Red orange single crystals suitable for X-ray diffraction analysis were obtained by slow evaporation from a 1:1 petroleum ether and ethyl acetate mixture.

### Refinement

All H atoms were placed at calculated positions and refined using a riding model approximation, with C—H = 0.93–0.97 Å and with *U*_iso_(H)=1.2 (1.5 for methyl groups) times *U*_eq_(C). A distance of 0.86 Å was assumed for the N3—H3A bond.

### Crystal data

**Table:**

C_21_H_19_ClF_3_N_3_O_2_	*D*_x_ = 1.436 Mg m^−^^3^
*M**_r_* = 437.84	Melting point = 423–425 K
Orthorhombic, *P*2_1_2_1_2_1_	Mo *K*α radiation, λ = 0.71073 Å
*a* = 7.940 (6) Å	Cell parameters from 3770 reflections
*b* = 12.602 (9) Å	θ = 2.6–24.7°
*c* = 20.233 (15) Å	µ = 0.24 mm^−^^1^
*V* = 2025 (3) Å^3^	*T* = 296 K
*Z* = 4	Block, orange
*F*(000) = 904	0.20 × 0.20 × 0.18 mm

### Data collection

**Table:**

Bruker APEXII CCD diffractometer	3576 independent reflections
Radiation source: fine-focus sealed tube	3162 reflections with *I* > 2σ(*I*)
graphite	*R*_int_ = 0.023
φ and ω scans	θ_max_ = 25.1°, θ_min_ = 1.9°
Absorption correction: multi-scan (*SADABS*; Bruker, 2001)	*h* = −9→9
*T*_min_ = 0.954, *T*_max_ = 0.958	*k* = −15→9
10369 measured reflections	*l* = −24→24

### Refinement

**Table:**

Refinement on *F*^2^	Secondary atom site location: difference Fourier map
Least-squares matrix: full	Hydrogen site location: inferred from neighbouring sites
*R*[*F*^2^ > 2σ(*F*^2^)] = 0.032	H-atom parameters constrained
*wR*(*F*^2^) = 0.078	*w* = 1/[σ^2^(*F*_o_^2^) + (0.0416*P*)^2^ + 0.1045*P*] where *P* = (*F*_o_^2^ + 2*F*_c_^2^)/3
*S* = 1.03	(Δ/σ)_max_ = 0.001
3576 reflections	Δρ_max_ = 0.15 e Å^−^^3^
273 parameters	Δρ_min_ = −0.21 e Å^−^^3^
0 restraints	Absolute structure: Flack (1983), 1511 Friedel pairs
Primary atom site location: structure-invariant direct methods	Flack parameter: −0.09 (6)

### Special details

**Table:**

Geometry. All e.s.d.'s (except the e.s.d. in the dihedral angle between two l.s. planes) are estimated using the full covariance matrix. The cell e.s.d.'s are taken into account individually in the estimation of e.s.d.'s in distances, angles and torsion angles; correlations between e.s.d.'s in cell parameters are only used when they are defined by crystal symmetry. An approximate (isotropic) treatment of cell e.s.d.'s is used for estimating e.s.d.'s involving l.s. planes.
Refinement. Refinement of *F*^2^ against ALL reflections. The weighted *R*-factor *wR* and goodness of fit *S* are based on *F*^2^, conventional *R*-factors *R* are based on *F*, with *F* set to zero for negative *F*^2^. The threshold expression of *F*^2^ > σ(*F*^2^) is used only for calculating *R*-factors(gt) *etc*. and is not relevant to the choice of reflections for refinement. *R*-factors based on *F*^2^ are statistically about twice as large as those based on *F*, and *R*- factors based on ALL data will be even larger.

### Fractional atomic coordinates and isotropic or equivalent isotropic displacement parameters (Å^2^)

**Table:**

	*x*	*y*	*z*	*U*_iso_*/*U*_eq_	
C1	−0.0571 (3)	0.51038 (15)	0.15673 (9)	0.0426 (5)	
C2	−0.0012 (3)	0.46256 (15)	0.09683 (10)	0.0479 (5)	
H2	0.0292	0.3913	0.0970	0.058*	
C3	0.0088 (3)	0.51828 (14)	0.03965 (10)	0.0450 (5)	
H3	0.0447	0.4842	0.0014	0.054*	
C4	−0.0343 (2)	0.62689 (14)	0.03692 (9)	0.0382 (4)	
C5	−0.0249 (2)	0.69109 (14)	−0.01980 (10)	0.0391 (4)	
H5	0.0082	0.6604	−0.0595	0.047*	
C6	−0.0629 (2)	0.79729 (14)	−0.01872 (9)	0.0358 (4)	
C7	−0.1134 (3)	0.84426 (14)	0.04343 (9)	0.0392 (4)	
C8	−0.0900 (2)	0.67227 (13)	0.09569 (9)	0.0366 (4)	
C9	−0.1056 (3)	0.61729 (14)	0.15382 (9)	0.0415 (5)	
H9	−0.1481	0.6508	0.1912	0.050*	
C10	−0.0636 (2)	0.86150 (14)	−0.08005 (8)	0.0363 (4)	
C11	0.1033 (2)	1.12141 (14)	−0.06355 (9)	0.0382 (4)	
C12	0.1722 (3)	1.18859 (16)	−0.01670 (10)	0.0471 (5)	
C13	0.2242 (3)	1.29033 (17)	−0.03257 (12)	0.0564 (6)	
H13	0.2701	1.3341	−0.0003	0.068*	
C14	0.2075 (3)	1.32630 (17)	−0.09657 (12)	0.0613 (6)	
H14	0.2430	1.3942	−0.1080	0.074*	
C15	0.1377 (3)	1.26061 (16)	−0.14320 (11)	0.0559 (6)	
H15	0.1242	1.2851	−0.1862	0.067*	
C16	0.0874 (3)	1.15921 (15)	−0.12760 (10)	0.0456 (5)	
H16	0.0424	1.1157	−0.1603	0.055*	
C17	−0.1226 (3)	0.80735 (15)	−0.14206 (10)	0.0434 (5)	
C18	0.0107 (3)	0.34867 (17)	0.21991 (12)	0.0607 (6)	
H18A	−0.0171	0.3080	0.1807	0.073*	
H18B	−0.0374	0.3124	0.2578	0.073*	
C19	0.1987 (3)	0.3531 (2)	0.22744 (16)	0.0921 (9)	
H19A	0.2465	0.3907	0.1907	0.138*	
H19B	0.2431	0.2823	0.2287	0.138*	
H19C	0.2266	0.3892	0.2678	0.138*	
C20	−0.1199 (3)	0.50530 (18)	0.27618 (10)	0.0596 (6)	
H20A	−0.0763	0.5772	0.2778	0.071*	
H20B	−0.0738	0.4667	0.3135	0.071*	
C21	−0.3098 (3)	0.50846 (19)	0.28210 (12)	0.0698 (7)	
H21A	−0.3558	0.5489	0.2462	0.105*	
H21B	−0.3405	0.5411	0.3233	0.105*	
H21C	−0.3535	0.4375	0.2807	0.105*	
Cl1	0.19498 (10)	1.14505 (5)	0.06447 (3)	0.0753 (2)	
F1	−0.24651 (16)	0.73715 (9)	−0.13101 (6)	0.0567 (3)	
F2	0.00093 (17)	0.75115 (10)	−0.17132 (6)	0.0632 (4)	
F3	−0.17985 (19)	0.87363 (10)	−0.18810 (5)	0.0661 (4)	
N1	−0.0641 (2)	0.45457 (13)	0.21455 (9)	0.0514 (4)	
N2	−0.0139 (2)	0.95674 (12)	−0.09268 (8)	0.0397 (4)	
N3	0.0552 (2)	1.01831 (12)	−0.04584 (8)	0.0437 (4)	
H3A	0.0694	0.9952	−0.0062	0.052*	
O1	−0.12920 (18)	0.77888 (9)	0.09745 (6)	0.0415 (3)	
O2	−0.1471 (2)	0.93702 (10)	0.05297 (6)	0.0522 (4)	

### Atomic displacement parameters (Å^2^)

**Table:**

	*U*^11^	*U*^22^	*U*^33^	*U*^12^	*U*^13^	*U*^23^
C1	0.0369 (11)	0.0411 (11)	0.0499 (11)	−0.0028 (9)	−0.0021 (9)	0.0060 (9)
C2	0.0502 (13)	0.0323 (10)	0.0613 (12)	0.0059 (10)	0.0028 (11)	0.0016 (9)
C3	0.0496 (12)	0.0335 (10)	0.0520 (11)	0.0050 (9)	0.0082 (10)	−0.0049 (9)
C4	0.0382 (11)	0.0322 (9)	0.0441 (10)	0.0007 (8)	0.0012 (8)	−0.0024 (8)
C5	0.0382 (11)	0.0361 (10)	0.0430 (10)	0.0023 (8)	0.0020 (9)	−0.0069 (8)
C6	0.0337 (10)	0.0334 (9)	0.0404 (10)	−0.0007 (8)	0.0015 (8)	−0.0041 (8)
C7	0.0395 (11)	0.0338 (10)	0.0441 (10)	0.0006 (9)	0.0034 (9)	−0.0006 (8)
C8	0.0356 (11)	0.0283 (9)	0.0458 (11)	0.0003 (8)	−0.0020 (9)	−0.0039 (8)
C9	0.0448 (12)	0.0373 (10)	0.0423 (11)	−0.0002 (9)	0.0035 (9)	−0.0008 (8)
C10	0.0359 (11)	0.0332 (10)	0.0398 (10)	0.0001 (8)	−0.0001 (8)	−0.0045 (8)
C11	0.0341 (11)	0.0338 (9)	0.0467 (10)	0.0001 (8)	0.0010 (9)	−0.0045 (9)
C12	0.0439 (12)	0.0480 (12)	0.0493 (11)	−0.0006 (9)	−0.0033 (10)	−0.0109 (9)
C13	0.0489 (14)	0.0448 (12)	0.0755 (15)	−0.0057 (11)	0.0002 (12)	−0.0199 (11)
C14	0.0652 (17)	0.0379 (11)	0.0809 (16)	−0.0099 (11)	0.0106 (14)	−0.0033 (12)
C15	0.0653 (15)	0.0442 (12)	0.0584 (12)	−0.0021 (11)	0.0072 (12)	0.0072 (11)
C16	0.0494 (12)	0.0402 (11)	0.0470 (11)	−0.0022 (9)	0.0002 (10)	−0.0045 (9)
C17	0.0439 (12)	0.0416 (10)	0.0448 (11)	0.0006 (10)	0.0007 (10)	−0.0035 (9)
C18	0.0596 (15)	0.0501 (13)	0.0723 (14)	0.0051 (12)	0.0076 (13)	0.0251 (12)
C19	0.0581 (18)	0.098 (2)	0.120 (2)	0.0182 (16)	0.0011 (17)	0.0512 (19)
C20	0.0794 (18)	0.0557 (13)	0.0437 (12)	0.0008 (13)	−0.0060 (11)	0.0080 (10)
C21	0.0807 (19)	0.0722 (16)	0.0566 (14)	0.0012 (15)	0.0098 (13)	0.0013 (12)
Cl1	0.0938 (5)	0.0819 (4)	0.0501 (3)	−0.0075 (4)	−0.0206 (3)	−0.0080 (3)
F1	0.0522 (7)	0.0562 (7)	0.0616 (7)	−0.0130 (6)	−0.0040 (6)	−0.0129 (6)
F2	0.0581 (8)	0.0710 (8)	0.0604 (7)	0.0048 (7)	0.0106 (6)	−0.0272 (6)
F3	0.0926 (11)	0.0582 (8)	0.0474 (7)	0.0008 (7)	−0.0190 (7)	−0.0007 (6)
N1	0.0567 (12)	0.0434 (9)	0.0542 (10)	0.0047 (9)	0.0003 (9)	0.0121 (8)
N2	0.0408 (10)	0.0356 (9)	0.0429 (8)	0.0006 (7)	0.0006 (7)	−0.0048 (7)
N3	0.0537 (11)	0.0375 (8)	0.0398 (9)	−0.0064 (8)	−0.0056 (7)	−0.0017 (7)
O1	0.0534 (9)	0.0311 (6)	0.0401 (7)	0.0056 (6)	0.0076 (6)	−0.0014 (6)
O2	0.0747 (11)	0.0300 (7)	0.0519 (8)	0.0090 (7)	0.0138 (8)	−0.0026 (6)

### Geometric parameters (Å, °)

**Table:**

C1—N1	1.366 (3)	C13—H13	0.9300
C1—C9	1.402 (3)	C14—C15	1.372 (3)
C1—C2	1.424 (3)	C14—H14	0.9300
C2—C3	1.356 (3)	C15—C16	1.376 (3)
C2—H2	0.9300	C15—H15	0.9300
C3—C4	1.412 (3)	C16—H16	0.9300
C3—H3	0.9300	C17—F3	1.331 (2)
C4—C8	1.392 (3)	C17—F1	1.342 (2)
C4—C5	1.406 (3)	C17—F2	1.347 (2)
C5—C6	1.372 (3)	C18—N1	1.465 (3)
C5—H5	0.9300	C18—C19	1.501 (4)
C6—C7	1.447 (3)	C18—H18A	0.9700
C6—C10	1.481 (3)	C18—H18B	0.9700
C7—O2	1.215 (2)	C19—H19A	0.9600
C7—O1	1.374 (2)	C19—H19B	0.9600
C8—C9	1.371 (3)	C19—H19C	0.9600
C8—O1	1.380 (2)	C20—N1	1.470 (3)
C9—H9	0.9300	C20—C21	1.513 (4)
C10—N2	1.289 (2)	C20—H20A	0.9700
C10—C17	1.503 (3)	C20—H20B	0.9700
C11—C12	1.384 (3)	C21—H21A	0.9600
C11—C16	1.387 (3)	C21—H21B	0.9600
C11—N3	1.401 (2)	C21—H21C	0.9600
C12—C13	1.385 (3)	N2—N3	1.342 (2)
C12—Cl1	1.741 (2)	N3—H3A	0.8600
C13—C14	1.378 (3)		
			
N1—C1—C9	121.30 (18)	C14—C15—H15	119.3
N1—C1—C2	121.56 (18)	C16—C15—H15	119.3
C9—C1—C2	117.14 (17)	C15—C16—C11	120.48 (19)
C3—C2—C1	121.68 (18)	C15—C16—H16	119.8
C3—C2—H2	119.2	C11—C16—H16	119.8
C1—C2—H2	119.2	F3—C17—F1	106.26 (17)
C2—C3—C4	121.42 (17)	F3—C17—F2	105.71 (17)
C2—C3—H3	119.3	F1—C17—F2	105.10 (15)
C4—C3—H3	119.3	F3—C17—C10	113.93 (16)
C8—C4—C5	118.53 (16)	F1—C17—C10	112.88 (16)
C8—C4—C3	116.23 (16)	F2—C17—C10	112.26 (17)
C5—C4—C3	125.24 (17)	N1—C18—C19	112.1 (2)
C6—C5—C4	122.46 (18)	N1—C18—H18A	109.2
C6—C5—H5	118.8	C19—C18—H18A	109.2
C4—C5—H5	118.8	N1—C18—H18B	109.2
C5—C6—C7	118.29 (18)	C19—C18—H18B	109.2
C5—C6—C10	121.36 (17)	H18A—C18—H18B	107.9
C7—C6—C10	120.22 (16)	C18—C19—H19A	109.5
O2—C7—O1	115.49 (16)	C18—C19—H19B	109.5
O2—C7—C6	126.37 (17)	H19A—C19—H19B	109.5
O1—C7—C6	118.12 (15)	C18—C19—H19C	109.5
C9—C8—O1	116.72 (16)	H19A—C19—H19C	109.5
C9—C8—C4	123.65 (17)	H19B—C19—H19C	109.5
O1—C8—C4	119.61 (16)	N1—C20—C21	112.3 (2)
C8—C9—C1	119.79 (17)	N1—C20—H20A	109.1
C8—C9—H9	120.1	C21—C20—H20A	109.1
C1—C9—H9	120.1	N1—C20—H20B	109.1
N2—C10—C6	132.33 (16)	C21—C20—H20B	109.1
N2—C10—C17	110.65 (16)	H20A—C20—H20B	107.9
C6—C10—C17	116.90 (16)	C20—C21—H21A	109.5
C12—C11—C16	117.78 (18)	C20—C21—H21B	109.5
C12—C11—N3	120.01 (18)	H21A—C21—H21B	109.5
C16—C11—N3	122.20 (17)	C20—C21—H21C	109.5
C11—C12—C13	121.7 (2)	H21A—C21—H21C	109.5
C11—C12—Cl1	119.64 (16)	H21B—C21—H21C	109.5
C13—C12—Cl1	118.65 (17)	C1—N1—C18	121.08 (18)
C14—C13—C12	119.6 (2)	C1—N1—C20	120.98 (17)
C14—C13—H13	120.2	C18—N1—C20	117.11 (18)
C12—C13—H13	120.2	C10—N2—N3	121.57 (16)
C15—C14—C13	119.1 (2)	N2—N3—C11	117.85 (16)
C15—C14—H14	120.4	N2—N3—H3A	121.1
C13—C14—H14	120.4	C11—N3—H3A	121.1
C14—C15—C16	121.3 (2)	C7—O1—C8	122.86 (14)
			
N1—C1—C2—C3	−178.8 (2)	Cl1—C12—C13—C14	179.90 (18)
C9—C1—C2—C3	1.5 (3)	C12—C13—C14—C15	0.7 (4)
C1—C2—C3—C4	0.7 (3)	C13—C14—C15—C16	−1.3 (4)
C2—C3—C4—C8	−1.1 (3)	C14—C15—C16—C11	1.2 (3)
C2—C3—C4—C5	178.8 (2)	C12—C11—C16—C15	−0.5 (3)
C8—C4—C5—C6	2.4 (3)	N3—C11—C16—C15	−179.3 (2)
C3—C4—C5—C6	−177.6 (2)	N2—C10—C17—F3	−27.1 (2)
C4—C5—C6—C7	0.3 (3)	C6—C10—C17—F3	156.38 (17)
C4—C5—C6—C10	−175.63 (17)	N2—C10—C17—F1	−148.43 (17)
C5—C6—C7—O2	178.2 (2)	C6—C10—C17—F1	35.1 (2)
C10—C6—C7—O2	−5.8 (3)	N2—C10—C17—F2	93.0 (2)
C5—C6—C7—O1	−3.3 (3)	C6—C10—C17—F2	−83.5 (2)
C10—C6—C7—O1	172.71 (16)	C9—C1—N1—C18	−170.4 (2)
C5—C4—C8—C9	179.41 (19)	C2—C1—N1—C18	9.9 (3)
C3—C4—C8—C9	−0.6 (3)	C9—C1—N1—C20	−1.1 (3)
C5—C4—C8—O1	−2.0 (3)	C2—C1—N1—C20	179.2 (2)
C3—C4—C8—O1	177.93 (18)	C19—C18—N1—C1	77.9 (3)
O1—C8—C9—C1	−175.76 (18)	C19—C18—N1—C20	−91.8 (3)
C4—C8—C9—C1	2.8 (3)	C21—C20—N1—C1	82.7 (3)
N1—C1—C9—C8	177.09 (18)	C21—C20—N1—C18	−107.6 (2)
C2—C1—C9—C8	−3.2 (3)	C6—C10—N2—N3	0.4 (3)
C5—C6—C10—N2	−140.5 (2)	C17—C10—N2—N3	−175.39 (17)
C7—C6—C10—N2	43.6 (3)	C10—N2—N3—C11	−178.98 (17)
C5—C6—C10—C17	35.1 (3)	C12—C11—N3—N2	178.80 (17)
C7—C6—C10—C17	−140.80 (19)	C16—C11—N3—N2	−2.4 (3)
C16—C11—C12—C13	−0.1 (3)	O2—C7—O1—C8	−177.57 (17)
N3—C11—C12—C13	178.75 (19)	C6—C7—O1—C8	3.7 (3)
C16—C11—C12—Cl1	−179.98 (15)	C9—C8—O1—C7	177.61 (18)
N3—C11—C12—Cl1	−1.2 (3)	C4—C8—O1—C7	−1.1 (3)
C11—C12—C13—C14	0.0 (3)		

### Hydrogen-bond geometry (Å, °)

**Table:**

*D*—H···*A*	*D*—H	H···*A*	*D*···*A*	*D*—H···*A*
N3—H3A···Cl1	0.86	2.57	2.960 (2)	109.
N3—H3A···O2	0.86	2.22	2.761 (3)	121.
C14—H14···O2^i^	0.93	2.55	3.316 (3)	140.

Symmetry codes: (i) *x*+1/2, −*y*+5/2, −*z*.
